# Radiosensitizing effects of miR‐18a‐5p on lung cancer stem‐like cells via downregulating both ATM and HIF‐1α

**DOI:** 10.1002/cam4.1527

**Published:** 2018-06-02

**Authors:** Xu Chen, Lei Wu, Dezhi Li, Yanmei Xu, Luping Zhang, Kai Niu, Rui Kong, Jiaoyang Gu, Zihan Xu, Zhengtang Chen, Jianguo Sun

**Affiliations:** ^1^ Cancer Institute, Xinqiao Hospital Army Medical University Chongqing China; ^2^ Department of Gerontology Chongqing General Hospital Chongqing China; ^3^ Oncology Department Leshan People’s Hospital Sichuan China

**Keywords:** DNA repair, lung adenocarcinoma, microRNAs, plasma biomarker, radiosensitivity, stem‐like cells

## Abstract

Lung cancer is one of the main causes of cancer mortality globally. Most patients received radiotherapy during the course of disease. However, radioresistance generally occurs in the majority of these patients, leading to poor curative effect, and the underlying mechanism remains unclear. In the present study, miR‐18a‐5p expression was downregulated in irradiated lung cancer cells. Overexpression of miR‐18a‐5p increased the radiosensitivity of lung cancer cells and inhibited the growth of A549 xenografts after radiation exposure. Dual luciferase report system and miR‐18a‐5p overexpression identified ataxia telangiectasia mutated (ATM) and hypoxia inducible factor 1 alpha (HIF‐1α) as the targets of miR‐18a‐5p. The mRNA and protein expressions of ATM and HIF‐1α were dramatically downregulated by miR‐18a‐5p in vitro and in vivo. Clinically, plasma miR‐18a‐5p expression was significantly higher in radiosensitive than in radioresistant group (*P* < .001). The cutoff value of miR‐18a‐5p >2.28 was obtained from receiver operating characteristic (ROC) curve. The objective response rate (ORR) was significantly higher in miR‐18a‐5p‐high group than in miR‐18a‐5p‐low group (*P* < .001). A tendency demonstrated that the median local progression‐free survival (PFS) from radiotherapy was longer in miR‐18a‐5p‐high than in miR‐18a‐5p‐low group (*P* = .082). The median overall survival (OS) from radiotherapy was numerically longer in miR‐18a‐5p‐high than in miR‐18a‐5p‐low group (*P* = .281). The sensitivity and specificity of plasma miR‐18a‐5p to predict radiosensitivity was 87% and 95%, respectively. Collectively, these results indicate that miR‐18a‐5p increases the radiosensitivity in lung cancer cells and CD133^+^ stem‐like cells via downregulating ATM and HIF‐1α expressions. Plasma miR‐18a‐5p would be an available indicator of radiosensitivity in lung cancer patients.

## 1 INTRODUCTION

Lung cancer is the most common type of malignancy and the leading cause of cancer‐related death globally. The five‐year survival rate of lung cancer is less than 20%.^1^, ^2^ Nonsmall cell lung cancer (NSCLC) accounts for 80%‐85% of all lung cancer subtypes, about 70% of which receives radiotherapy during the whole period of disease. However, most NSCLC patients suffer from radioresistance, leading to poor therapeutic outcomes.^3^ The mechanism underlying radioresistance is so complicated that it remains unclear despite decades of efforts by radiobiological scientists and oncologist worldwide.

Cancer stem‐like cells play an important role in tumorigenesis, tumor invasion, metastasis, and recurrence.^4^ Increasing studies manifest that cancer stem‐like cells are highly related to radioresistance and have become a hotspot in this field.^5^, ^6^, ^7^, ^8^ Thus, it is urgent to explore the role of cancer stem‐like cells in radioresistance and the underlying regulatory mechanism in lung cancer.

MicroRNAs (miRNAs) are a kind of noncoding small RNAs that usually downregulate the targets via binding to the 3′‐UTR of mRNAs. The expression of miRNAs keeps stable in tissues, cells, plasma, urine, etc.^9^, ^10^, ^11^ The miRBase 21^12^ shows that there are only 1881 human miRNAs, regulating one‐third human genes. Usually, one miRNA regulates multiple targets and could be regulated by multiple genes.^13^ It has been reported that miRNAs are involved in tumorigenesis, cell cycle, cell proliferation, apoptosis, tumor microenviroment, and radioresistance, acting as tumor suppressors or tumor promoters.^14^, ^15^, ^16^, ^17^, ^18^, ^19^, ^20^ It has also been verified that miRNAs are related to biological characteristics of cancer stem‐like cells, such as self‐renewal, proliferation, invasion, and radiotherapy or chemotherapy resistance.^21^, ^22^

Our previous study demonstrated that miR‐18a‐5p was downregulated in CD133^+^ stem‐like cells in lung cancer compared with CD133^−^ cells.^23^ Bioinformatic analysis suggested that ataxia telangiectasia mutated (ATM) related to DNA repair and hypoxia inducible factor 1 alpha (HIF‐1, HIF‐1α) related to hypoxia in tumor microenvironment are both potential targets of miR‐18a‐5p. Hence, in the current study, we explored whether miR‐18a‐5p would affect the radiosensitivity of cancer stem‐like cells in NSCLC.

## 2 MATERIALS AND METHODS

### 2.1 Cell culture

Three cell lines, human bronchial epithelial cell line (HBE), lung adenocarcinoma A549, and SPC‐A1 cell lines were kept in our institute. HBE, A549, and SPC‐A1 cells were cultured in RPMI‐1640 medium (Hyclone) supplemented with 10% FBS (Hyclone) and 1% Penicillin‐Streptomycin Solution (Hyclone) in an incubator with 5% CO_2_ at 37°C. The CD133^+^ stem‐like cells were induced and then isolated from the A549 cells as described in our previous study.^23^ CD133^+^ and CD133^−^ cells were sorted by flow cytometry (FCM) and used in subsequent experiments.

### 2.2 Quantitative real‐time PCR (qRT‐PCR)

The total RNA of cancer cells was extracted and used for reverse transcription according to the instructions of PrimeScript^™^ RT Reagent Kit (#RR037A, TaKaRa). The real‐time PCR was performed using SYBR^®^ Premix Ex Taq^™^ II (#RR820A, TaKaRa). The reaction condition was as follows: 95°C for 5 seconds, 40 cycles of 95°C for 30 seconds, and 60°C for 34 seconds. U6 small nuclear RNA (U6 snRNA) and β‐actin were used as an internal control for miRNAs and mRNA amplification, respectively. The reverse transcription primer of miR‐18a‐5p was 5′‐gtcgtatccagtgcagggtccgaggtattcgcactggatacgacctatct‐3′. All of the sense and antisense primers in qRT‐PCR assay are listed in Table 1. The qRT‐PCR reactions were repeated three times.

**Table 1:** qRT‐PCR primers for miRNAs and mRNAs

	Sense primer	Antisense primer
miR‐18a‐5p	5′‐acgtaaggtgcatctagtgcagata‐3′	5′‐gtgcagggtccgaggt‐3′
U6 snRNA	5′‐ctcgcttcggcagcaca‐3′	5′‐aacgcttcacgaatttgcgt‐3′
cel‐39	5′‐gtcgtatccagtgcagggtccgaggtattcgcactggatacgaccaagct‐3′	5′‐tcaccgggtgtaaatcagctt‐3′
Oct4	5′‐aagctgctgaaacagaagagg‐3′	5′‐acacggttctcaatgctagtc‐3′
Nestin	5′‐gcaaaggagcctactccaag‐3′	5′‐agatggagcaggcaagagat‐3′
Nanog	5′‐atttgcggccgcatgagtgtgggtcttc‐3′	5′‐cgggatcctcatatttcacctggtggag‐3′
ATM	5′‐gttgccaaggtagctcagtct‐3′	5′‐ctggctcccctatacttctgtag‐3′
HIF‐1α	5′‐caccgattcgccatgga‐3′	5′‐tttcttttcgacgttcagaactcat‐3′
β‐actin	5′‐gcgagcacagagcctcgcctt‐3′	5′‐catcatccatggtgagctggcgg‐3′

### 2.3 Bioinformatic analysis

Three online software programs including PicTar,^24^ TargetScan,^25^ and miRanda^26^ were used to predict the potential targets of miR‐18a‐5p. The potential binding sites between miR‐18a‐5p and the 3′‐UTR of ataxia telangiectasia mutated (ATM) and hypoxia inducible factor 1 alpha (HIF‐1α) were obtained from PicTar program.

### 2.4 Dual luciferase reporter assay

Through searching in NCBI GenBank database, 220‐240 nt 3′‐UTR of targets (100 nt upstream and downstream from binding site) were synthesized with or without seed sequence (named 3UTR‐WT and 3UTR‐MUT, respectively). The dangling ends of synthesized fragments were added with Xba I restriction site (TCTAGA). The modified fragments were inserted into dual luciferase reporter vector pGV306 (GeneChem, Shanghai, China), respectively. In dual luciferase reporter assay, miRNA mimics negative control (miR‐NC) was also used. The vectors were divided into four groups: Test group (pGV306‐3UTR‐WT plus miR‐18a‐5p mimics), Con‐1 group (pGV306‐3UTR‐WT plus miR‐NC), Con‐2 group (pGV306‐3UTR‐MUT plus miR‐18a‐5p mimics), and Con‐3 group (pGV306‐3UTR‐MUT plus miR‐NC). When the confluence reached 80%‐90% in a 24‐well plate, cotransfection reaction contained 500 ng of recombinant pGV306, and 2.5 μL of miR‐18a‐5p mimic or miR‐NC in 500 μL after addition of lipofectamine 2000 (Invitrogen, Carlsbad, CA). The final concentration of miR‐18a‐5p mimic or miR‐NC was 30 nmol/L. Transfected cells were collected 24 hours later and luciferase activity was analyzed by a dual‐luciferase reporter system (Promega, Madison, WI). The luciferase ratio of Firefly/Renilla represented the expressions of targets. The data of each group are presented as mean ± standard deviation (SD).

### 2.5 Western blotting

The total protein of the cancer cells and xenografts was extracted according to the manufacturer’s instruction (Sigma‐Aldrich, St. Louis, MO). During the extraction, protease inhibitor (100:1) and phosphorylated protease inhibitor (100:1) were used. Western blotting was performed as described in detail in our early report 27 using the primary antibodies of ATM, pATM (phospho S1981), HIF‐1α, Lamin B, and γH2AX, GAPDH (Proteintech Group, China) and secondary antibodies (BOSTER, China). The concentration of all primary and secondary antibodies was 1:5000 except that of GAPDH (1:1000). In the HIF‐1α detection, CoCl_2_ was used to induce hypoxia to avoid the degradation of HIF‐1α.

### 2.6 miR‐18a‐5p overexpression in SPC‐A1, A549, and CD133^+^ cells

To overexpress miR‐18a‐5p in SPC‐A1 and A549 cells, miRNA mimics (Ribobio, China) at different concentrations (0.1, 0.2 or 0.3 μmol/L) were chosen to follow the instruction of siPORT^™^ NeoFX^™^ Transfection Agent (ABI, Vernon, CA). The culture medium was renewed 24 hours later. Then, the expressions of miR‐18a‐5p and potential targets were detected.

To overexpress miR‐18a‐5p in CD133^+^ stem‐like cells, a lentivirus system (GeneChem) was introduced. A recombinant lentivirus (miR‐18a‐5p‐LV) was generated by cloning full‐length human miR‐18a‐5p (LM378759) into the AgeI/NheI sites of lentivirus vector pGV369 (GeneChem, Shanghai, China) as previously described.^28^ Recombinant miR‐18a‐5p‐LV was verified by sequencing. Then miR‐18a‐5p‐LV and packaging plasmids were co‐transfected into HEK‐293T cells to produce lentiviral particles. Active lentiviral particles with a multiplicity of infection (MOI) value of 30 were mixed with CD133^+^ stem‐like cells supplemented with 5 μg/mL of polybrene (GeneChem, Shanghai, China), and seeded to an ultra‐low attachment 24‐well plate with 500 μL of enhanced infection solution (Eni.s). Eni.s was replaced by serum‐free medium supplemented with 0.4% BSA (Amresco), insulin (5 μg/mL, Sigma), basic fibroblast growth factor (bFGF, 10 ng/mL, PeproTech), and human recombinant epidermal growth factor (EGF, 20 ng/mL, PeproTech) 10 hours later. Another 48 hours later, the fluorescence of GFP was observed under an inverted microscope.

### 2.7 Clonogenic assay in 3D matrigel culture

Clonogenic assay in 3D matrigel culture was performed as described in our previous study.^27^ Briefly, CD133^+^ stem‐like cells were divided into three groups: control group (Blank), empty vector group (EV control), and miR‐18a‐5p group (miR‐18a‐5p‐LV). CD133^+^ stem‐like cells in exponential growth phase were digested into single cells, resuspended in IMDM containing 10% calf serum, and seeded in solidified matrigel in a 24‐well plate at a cell density of 200 cells per well. Then, the cells were cultured in an incubator with 5% CO_2_ at 37°C for 2 weeks. The colonies containing more than 50 cells were counted. There were six wells in each group in clonogenic assay, and the experiments were repeated three times.

### 2.8 Radiation survival curves and radiobiological parameters

A549 cells were divided into the following three groups: control group (Blank), negative mimics control group (miR‐NC), and miR‐18a‐5p group (miR‐18a‐5p mimics). Similarly, CD133^+^ stem‐like cells were divided into three groups: control group (Blank), empty vector group (EV control), and miR‐18a‐5p group (miR‐18a‐5p‐LV). A549 or CD133^+^ stem‐like cells in exponential growth phase were digested into single cells, resuspended at a cell density of 10^4^ cells/mL in IMDM containing 10% calf serum, and seeded into a 24‐well plate at 200, 400, 800, 2000, 5000, and 10 000 cells, respectively. Next, A549 or CD133^+^ cells were irradiated with 6 MV X‐ray at doses of 0, 2, 4, 6, 8, and 10 Gy, correspondingly. Then, the cells after irradiation exposure were cultured in an incubator with 5% CO_2_ at 37°C for 2 weeks. The culture medium was updated every 2 days. The colonies containing more than 50 cells were counted. Survival fraction (SF) was calculated from the formula SF = clonogenic ratio with radiation/clonogenic ratio without radiation. GraphPad Prism 4.0 was used to draw radiation survival curves (10 Gy dose point excluded because of too few colonies), and radiobiological parameters (D0, Dq and SF2) were calculated. Furthermore, sensitive enhancement ration (SER) was obtained from the formula SER = SF2_EV control_/SF2_miR‐18a‐5p‐LV_ at 2 Gy.

### 2.9 Animal experiments

Female nude mice aged 5‐6 weeks (Beijing HFK BioScience Co., LTD) were used for in vivo experiment. According to the above grouping of cells, mice bearing A549 cells were divided into three groups: control group (Blank), negative mimics control group (miR‐NC), and miR‐18a‐5p group (miR‐18a‐5p mimics). To construct the xenograft model, 1 × 10^6^ of A549 cells from each group (Blank, EV control and miR‐18a‐5p‐LV) were inoculated into the right leg of the mouse. When the tumor volume reached about 100 mm^3^, local irradiation of 6MV X‐ray was administered after anesthetization. The total dose was 20 Gy in 2 fractions with 10 Gy per fraction. Then the tumor volume was measured and calculated every 2 days. The tumor volume (*V*) was calculated according to the formula *V* = (length × width^2^)/2. The tumor samples were collected at the deadline of survival observation on day 30 or timepoint of morbidity.

### 2.10 Clinical study

Patients were selected and analyzed as described in our previous study.^29^ Briefly, patients with unresectable stage III or IV NSCLC according to International Classification of Diseases for Oncology 30 were enrolled from January 2013 to December 2014 (Table 2). After 2‐4 cycles of chemotherapy, thoracic radiotherapy to the planning gross tumor volume (pGTV) (60‐66 Gy/30‐33F/6‐7w) was given in patients with Eastern Cooperative Oncology Group (ECOG) performance status (PS) of 0‐2 and an age of 18‐75 years. This study has been approved by the Ethics Committee of our university. About 5 mL of noncoagulant blood was drawn before radiotherapy, and plasma miR‐18a‐5p was then detected by qRT‐PCR with synthetic cel‐miR‐39‐3p small RNA (cel‐39) as an external control.^29^ The primers of cel‐39 and miR‐18a‐5p are listed in Table 1. By the deadline of follow‐up on 31 October 2015, tumor response estimated by RECIST 1.1, the median local progression‐free survival (PFS) and overall survival (OS) from radiotherapy had been recorded. The objective response rate (ORR) was defined as the proportion of patients with complete response (CR) or partial response (PR). Adverse effects, mainly radiation pneumonia (RP), were also recorded.^29^

**Table 2:** Clinical characteristics of enrolled patients

Stratification factors	Total (n = 54)	Radiosensitive subgroups (n = 15)	Radioresistant subgroups (n = 39)	*P* value
Age (years)				
Median	60 (31‐75)	58.5 (31‐71)	60.6 (38‐75)	.503
Sex (%)				
Male	47 (87.0)	14 (93.3)	33 (84.6)	.688
Female	7 (13.0)	1 (6.7)	6 (15.4)	
Histology (%)				
Squamous	32 (59.3)	9 (60.0)	23 (59.0)	.945
Nonsquamous	22 (40.7)	6 (40.0)	16 (41.0)	
Stage (%)				
IIIa	7 (13.0)	2 (13.3)	5 (12.8)	.997
IIIb	22 (40.7)	6 (40.0)	16 (41.0)	
IV	25 (46.3)	7 (46.7)	18 (46.2)	
Smoking (%)				
No	17 (31.5)	4 (26.7)	13 (33.3)	.884
Yes	37 (68.5)	11 (73.3)	26 (66.7)	
Radiation pneumonia				
Yes	31 (57.4)	9 (60.0)	22 (56.4)	.811
No	23 (42.6)	6 (40.0)	17 (43.6)	

### 2.11 Statistical analysis

The expressions of plasma miR‐18a‐5p were compared by independent‐samples *t* test based on homogeneity test. The cu‐off value of miR‐18a‐5p relative to cel‐39 was obtained from receiver operating characteristic (ROC) curve using MedCalc 15.10. Based on the cutoff value, ORR comparison was realized by the standard chi‐square test to obtain predictive sensitivity and specificity of plasma miR‐18a‐5p. The median PFS and OS were estimated using the Kaplan‐Meier method. Statistical analysis was performed using SPSS statistical package (version 17.0, SPSS Inc., Chicago, IL), and statistical significance was set at *P* < .05.

## 3 RESULTS

### 3.1 Radiosensitizing effects of miR‐18a‐5p on A549 cells

**Figure 1:**
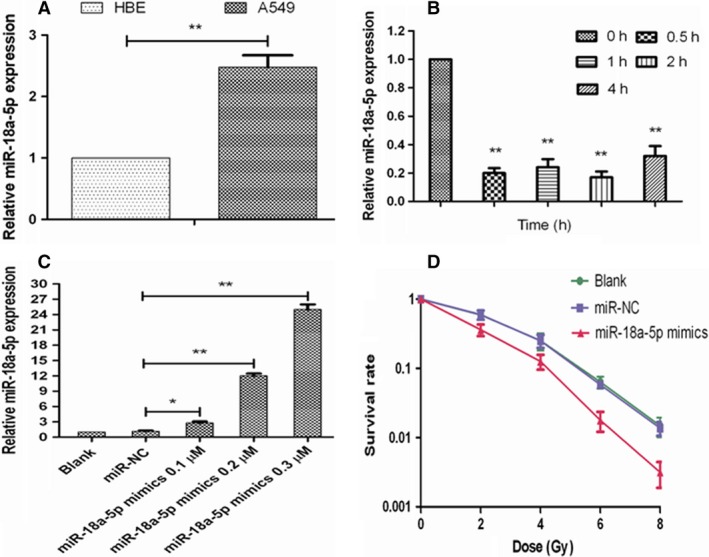
Radiosensitizing effects of miR‐18a‐5p on A549 cells. A, miR‐18a‐5p expression in HBE and A549 cells (**P < .01). B, miR‐18a‐5p expression in A549 cells before and after 2Gy X‐ray irradiation (**P < .01 vs 0 h). C, miR‐18a‐5p expression in A549 cells before and after miR‐18a‐5p mimics transfection (*P < .05, **P < .01). D, Radiation survival curves of A549 cells before and after miR‐18a‐5p mimics transfection

The expressions of miR‐18a‐5p in HBE and A549 cells before and after irradiation were detected by qRT‐PCR. The results showed that miR‐18a‐5p was more highly expressed in A549 cells than in HBE cells (Figure 1A). The radiation at a dose of 2 Gy continuously decreased the expression of miR‐18a‐5p in A549 cells at the time point of 0.5, 1, 2, and 4 hours to 0.2, 0.22, 0.18, and 0.31, nomalized to the expression in unirradiated A549 cells (*P* < .01, Figure 1B), which was similar in SPC‐A1 cells and the corresponding expressions were 0.25, 0.47, 0.27, and 0.65, respectively. All the concentrations of miR‐18a‐5p mimics (0.1, 0.2, and 0.3 μmol/L) significantly increased the expression of miR‐18a‐5p to 2.42, 10.37, and 21.61 times before in A549 cells (*P* < .01, Figure 1C). Then, the concentration of 0.2 μmol/L was used in subsequent experiments.

Radiation survival curves of A549 cells for different groups showed that the D0, Dq, and SF2 values in miR‐18a‐5p mimics group (1.70, 0.64, and 0.34) were significantly lower than those in blank group (1.75, 1.08, and 0.48) and miR‐NC group (1.75, 1.10, and 0.48). The SER was 1.41 (vs blank group) and 1.41 (vs miR‐NC group) (*P* < .05, Table 3). Also, radiation survival curves of SPC‐A1 cells for different groups demonstrated the similar results (data not shown). All the results indicated that miR‐18a‐5p overexpression radiosensitized A549 cells (Figure 1D).

**Table 3:** Radiobiological parameters in A549 and CD133^+^ cells (*P<.05)

Cells	Groups	D0(Gy)	Dq(Gy)	SF2	SER
A549	miR‐18a‐5p mimics	1.70*	0.64*	0.34*	‐
	miR‐NC	1.75	1.10	0.48	1.41
	Blank	1.75	1.08	0.48	1.41
CD133^+^	miR‐18a‐5p‐LV	0.84*	0.5*	0.16*	‐
	EV control	1.08	0.79	0.3	1.89
	Blank	1.09	0.77	0.3	1.85

### 3.2 Radiosensitizing effects of miR‐18a‐5p on CD133^+^ stem‐like cells

**Figure 2:**
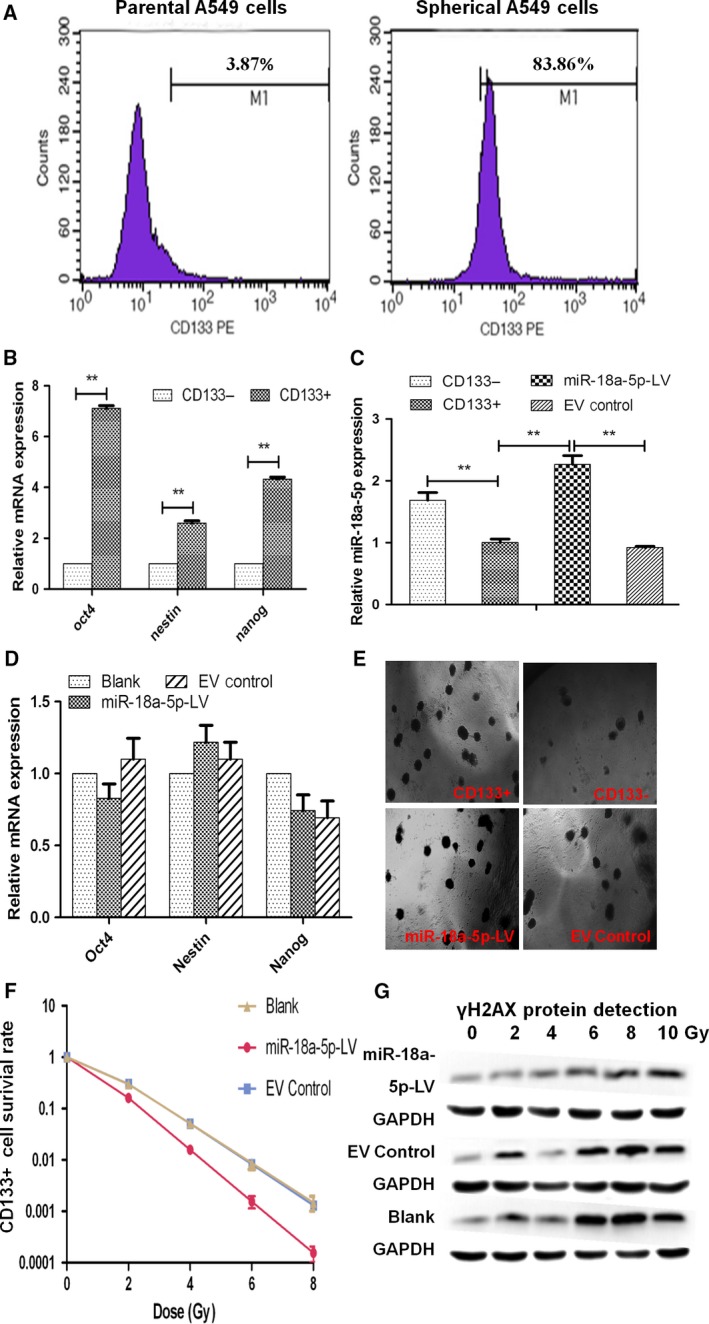
Radiosensitizing effects of miR‐18a‐5p on CD133^+^ cells. A, The proportion of CD133^+^ stem‐like cells in parent A549 cells and A549 cells induced into spheres. B, mRNA expression of stemness factors (Oct4, Nestin, Nanog) in CD133‐ and stem‐like CD133^+^ cells (**P < .01). C, miR‐18a‐5p expression in stem‐like CD133^+^ cells before and after miR‐18a‐5p‐LV infection (*P < .05, **P < .01). D, mRNA expression of stemness factors (Oct4, Nestin, Nanog) in stem‐like CD133^+^ cells before and after miR‐18a‐5p infection. E, Clonogenic assay in 3‐D matrigel in CD133^+^ cells with or without miR‐18a‐5p‐LV infection under magnification 40X (*P* < .01, n = 5). F, Radiation survival curves of stem‐like CD133^+^ cells before and after miR‐18a‐5p infection. G, the expression of γH2AX in CD133^+^ stem‐like cells with miR‐18a‐5p‐LV infection by Western blotting 30 min after irradiation

After being induced by paclitaxel and serum‐free medium, epithelial A549 cells turned into spheres in suspension status. The proportion of CD133^+^ stem‐like cells in parental A549 cells reached 3.87%, while the proportion of CD133^+^ stem‐like cells in these spheres under FCM reached about 83.86% (Figure 2A). CD133^+^ and CD133^−^ cells were sorted by FCM, and the purity of CD133^+^ cells reached 95.8%. Clonogenic assay in matrigel and nude mice was repeated to confirm the stemness of CD133^+^ cells as described in our previous study.^23^ In qRT‐PCR assay, CD133^+^ cells expressed stemness factors (Oct4, Nestin, and Nanog) significantly higher than CD133^−^ cells (*P* < .05, Figure 2B). Consistently, the expression of miR‐18a‐5p in CD133^+^ cells was 0.61 ± 0.08 times lower than that in CD133^−^ cells (*P* < .01, Figure 2C).

To investigate the biological function of miR‐18a‐5p, we introduced miR‐18a‐5p‐LV into CD133^+^ cells. After infection with miR‐18a‐5p‐LV, CD133^+^ stem‐like cells displayed GFP fluorescence at a ratio of 92% under a fluorescence microscope. EV control also reached an infection efficacy of 86%.

After infection, miR‐18a‐5p was expressed about 2.25 ± 0.25 times higher in miR‐18a‐5p‐LV group than in EV control group or blank group (*P* < .01, Figure 2C). To determine whether miR‐18a‐5p was related to self‐renewal of CD133^+^ cells, several experiments were performed. In qRT‐PCR assay, there was little change in stemness factors of Oct4, Nanog, or Nestin after miR‐18a‐5p overexpression compared with EV control group (*P* > .05, Figure 2D). Clonogenic assay in 3D matrigel culture showed that the clonogenic potential of parental CD133^+^ stem‐like cells was 34 ± 1%, compared with 12 ± 1% in CD133^−^ cells (*P* < .05, Figure 2E). After infection with miR‐18a‐5p‐LV, CD133^+^ stem‐like cells generated similar spheroids (29 ± 1%) to EV control (25 ± 1%), suggesting that miR‐18a‐5p did not affect the self‐renewal of CD133^+^ stem‐like cells (*P* > .05, n = 5, Figure 2E). Taken together, miR‐18a‐5p was not critical for stemness regulation of CD133^+^ stem‐like cells on sphere ability or stemness factors.

Radiation survival curves of CD133^+^ cells for different groups showed that the D0, Dq and SF2 values were significantly lower in miR‐18a‐5p‐LV group (0.84, 0.5 and 0.16) than in blank group (1.09, 0.77 and 0.3) and EV control group (1.08, 0.79 and 0.3). The SER was 1.85 (vs blank group) and 1.89 (vs EV control group) (*P* < .05, Table 3). The results indicated that the overexpression of miR‐18a‐5p enhanced the radiosensitivity of CD133^+^ stem‐like cells to X‐ray irradiation (Figure 2F).

Additionally, the expression of γH2AX was detected in CD133^+^ stem‐like cells with miR‐18a‐5p‐LV infection by Western blotting 30 minutes after irradiation. The expression of γH2AX protein increased with the radiation dose in a certain range in all these groups, while miR‐18a‐5p overexpression downregulated the expression of γH2AX protein (Figure 2G).

### 3.3 Verification of ATM and HIF‐1α as the targets of miR‐18a‐5p

Some potential targets of miR‐18a‐5p predicted by the online software programs are listed as follows: NEDD9, GLRB, INADL, ESR1, HMGCS1, TMEM2, RTP4A3, RAB9A, RORA, ATM, and HIF‐1α. They could be involved in oncogenesis, anti‐oncogenesis, transcription, DNA repair, cell cycle regulation, miRNA processing and signal transduction. Among them, ATM (associated with DNA repair) and HIF‐1α (associated with hypoxia in tumor microenvironment) are possibly correlated with radiosensitivity in A549 cells and CD133^+^ stem‐like cells. Free binding energy (FBE) cells analysis 31 showed that ATM and HIF‐1α could be the putative targets of miR‐18a‐5p. The binding free energy between ATM and miR‐18a‐5p was −22.8 kcal/mol, dramatically lower than average random free energy of ATM −2.92 kcal/mol obtained at website.^32^ The binding free energy between HIF‐1α and miR‐18a‐5p was −19.0 kcal/mol, dramatically lower than average random free energy of HIF‐1α ‐0.62 kcal/mol as well. Additionally, miR‐18a‐5p had highly conserved binding sites to ATM and HIF‐1α in discrepant species. Thus, ATM and HIF‐1α were chosen for further exploration.

**Figure 3:**
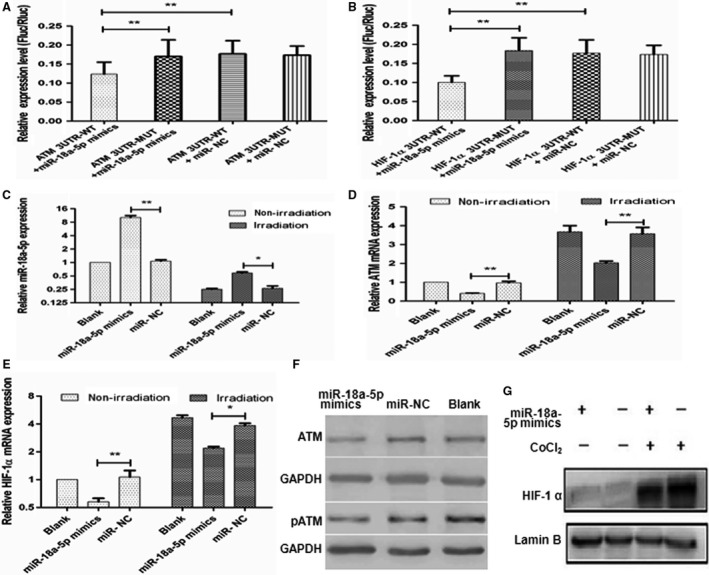
Verification of the targets of miR‐18a‐5p. A, Histogram of dual luciferase assay of ATM verification (**P < .01). B, Histogram of dual luciferase assay of HIF‐1α verification (**P < .01). C, miR‐18a‐5p expression in A549 cells before and after miR‐18a‐5p mimics transfection with or without 2 Gy X‐ray irradiation (*P < .05, **P < .01). D, ATM mRNA expression in A549 cells before and after miR‐18a‐5p mimics transfection with or without 2 Gy X‐ray irradiation (**P < .01). E, HIF‐1α mRNA expression in A549 cells before and after miR‐18a‐5p mimics transfection with or without 2 Gy X‐ray irradiation (*P < .05, **P < .01). F, ATM and pATM protein expression before and after miR‐18a‐5p mimics transfection in A549 cells. G, HIF‐1α protein expression after and before miR‐18a‐5p mimics transfection with or without CoCl_2_ in A549 cells

In the dual‐luciferase reporter assay to verify ATM as a target of miR‐18a‐5p, the relative luciferase activity in the test group was significantly lower than that in the blank and miR‐NC groups (*P* < .01, Figure 3A). Similarly, in the assay to verify HIF‐1α as a target of miR‐18a‐5p, the ratio of firefly luciferase to renilla in miR‐18a‐5p mimics group was significantly lower than that in the blank and miR‐NC groups (*P* < .01, Figure 3B). Thus, dual‐luciferase reporter assay indicated that miR‐18a‐5p could bind to the 3‐UTR of both ATM and HIF‐1α.

The correlation between miR‐18a‐5p expression and potential targets was analyzed at both mRNA and protein levels. The mRNA expressions of miR‐18a‐5p, ATM, and HIF‐1α in A549 cells with the transfection 0.2 μmol/L miR‐18a‐5p mimics were detected before and after 2 Gy X‐ray irradiation. Before irradiation, miR‐18a‐5p was expressed 12.04 times higher in miR‐18a‐5p mimics group than in miR‐NC group (*P* < .01, Figure 3C). The mRNA expression of ATM in miR‐18a‐5p mimics group was 0.42 times lower than in miR‐NC group before irradiation (*P* < .01, Figure 3D), and the mRNA expression of HIF‐1α in miR‐18a‐5p mimics group was 0.58 times lower than that in miR‐NC group before irradiation (*P* < .01, Figure 3E). After irradiation, the expression of miR‐18a‐5p in each group decreased. Accordingly, the mRNA expressions of ATM and HIF‐1α in each group increased. However, the expression of miR‐18a‐5p in miR‐18a‐5p mimics group was still 2.23 times higher than that in miR‐NC group (*P* < .05, Figure 3C). The mRNA expression of ATM in miR‐18a‐5p mimics group was 0.54 times lower than that in miR‐NC group after irradiation (*P* < .01, Figure 3D). The mRNA expression of HIF‐1α in miR‐18a‐5p mimics group was 0.58 times lower than that in miR‐NC group after irradiation (*P* < .05, Figure 3E).

In addition, the protein expressions of ATM and HIF‐1α with the transfection 0.2 μmol/L miR‐18a‐5p mimics were detected by Western blotting. The protein levels of both ATM and pATM (Phospho S1981) were downregulated by miR‐18a‐5p mimics in A549 cells compared with the miR‐NC group (Figure 3F). Likewise, in SPC‐A1 cells, miR‐18a‐5p mimics downregulated pATM protein to 72.9% compared with the miR‐NC group. To determine whether HIF‐1α protein was a target, CoCl_2_ was used to induce hypoxia before and after miR‐18a‐5p mimics transfection. The expression of HIF‐1α protein was very low under normal condition without CoCl_2_ treatment, but higher under hypoxic condition with CoCl_2_ treatment. Similarly, the protein level of HIF‐1α was dramatically lower in miR‐18a‐5p mimics group than in miR‐NC group (Figure 3G). Generally, the expression of miR‐18a‐5p was negatively correlated with the mRNA and protein levels of ATM and HIF‐1α in vitro.

### 3.4 Functional analysis of miR‐18a‐5p in animal experiments

**Figure 4:**
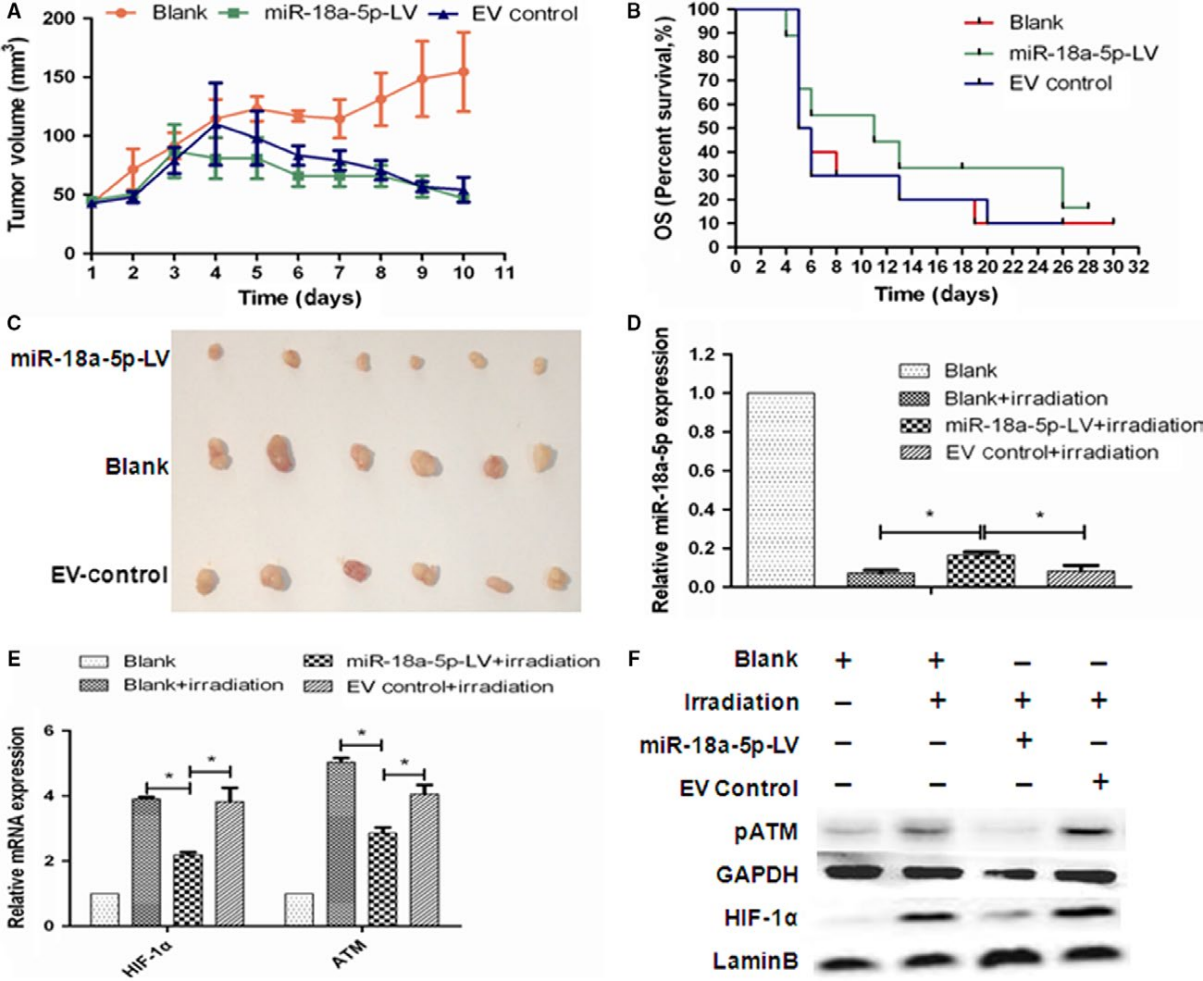
Radiosensitizing effects of miR‐18a‐5p in vivo under irradiation. A, Growth curves of A549 xenografts at day 10 (n = 6, *P* < .05). B, Survival time of experimental mice at day 30 (n = 6, *P* > .05). C, Tumor samples in A549 xenografts (n = 6, *P* < .05). D, miR‐18a expression in A549 xenografts (*P < .05). E, ATM and HIF‐1α mRNA expression in A549 xenografts (*P < .05). F, pATM and HIF‐1α protein expressions in A549 xenografts

There were 10 mice in each group. After inoculation, only one mouse showed no tumor in the miR‐18a‐5p‐LV group. We chose six mice bearing xenografts in similar size from each group to analyze the function of miR‐18a‐5p. The in vivo results demonstrated that compared with the EV vector and blank groups, tumor volume and tumor growth rate decreased in the miR‐18a‐5p‐LV group in the early 8 days (*P* = .012, Figure 4A). There was no significant difference in survival time on day 30 between the three groups (*P* > .05, Figure 4B). However, the tumor samples in the miR‐18a‐5p‐LV group were significantly smaller than those in the other two groups (*P* < .05, Figure 4C).

To analyze the impact of miR‐18a‐5p‐LV infection, the expressions of miR‐18a‐5p and ATM and HIF‐1α targets in xenografts were detected. In blank group without irradiation, the expression of miR‐18a‐5p was high in A549 xenograft. Under irradiation, the expression of miR‐18a‐5p in each group decreased at discrepant range. Basically, compared with the EV control and blank groups, the expression of miR‐18a was significantly increased in the miR‐18a‐5p‐LV group (*P* < .05, Figure 4D). Consistently, the mRNA expressions of ATM and HIF‐1α were low in A549 xenograft in blank group without irradiation. Under irradiation, the mRNA expressions of ATM and HIF‐1α in each group increased at discrepant range. In qRT‐PCR assay, the mRNA expressions of ATM and HIF‐1α under irradiation were significantly lower in the miR‐18a‐5p‐LV group than in the EV control and blank groups (*P* < .05, Figure 4E). In addition, Western blotting showed protein changes of pATM and HIF‐1α consistent with mRNA changes in each group (Figure 4F). Therefore, irradiation decreased miR‐18a‐5p but increased pATM and HIF‐1α subsequently, and miR‐18a‐5p inhibited pATM and HIF‐1α at both mRNA and protein levels in vivo, consistent with in vitro results.

### 3.5 Plasma miR‐18a‐5p as an indicator of radiosensitivity

A total of 54 patients were enrolled in our study, including 47 males and seven females with a median age of 60 years (range: 31‐75 years), 37 cases (68.5%) of ever or current smokers, 22 cases (40.7%) of adenocarcinoma, and 32 cases (59.3%) of squamous carcinoma. There were seven cases (13.0%) in stage IIIa with unresectable tumors, 22 (40.7%) cases in stage IIIb, and 25 cases (46.3%) in stage IV.

**Figure 5:**
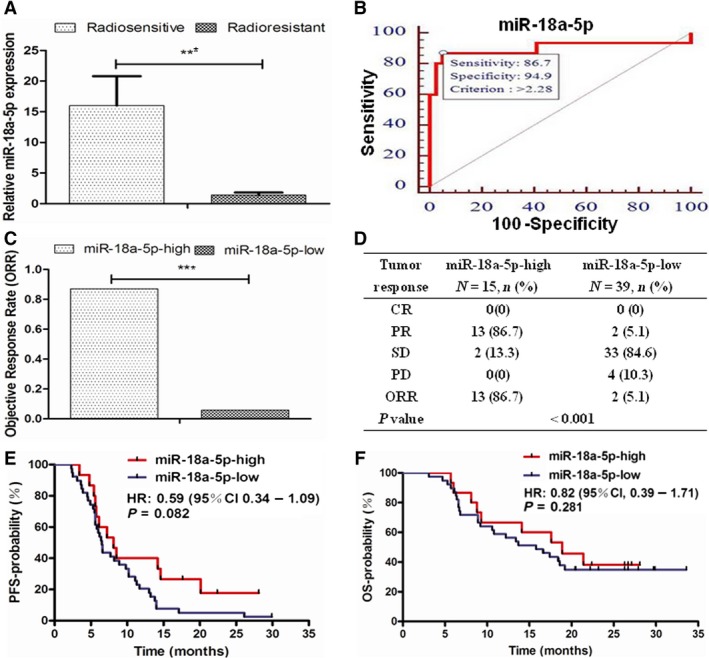
Plasma miR‐18a‐5p in patients. A, Plasma miR‐18a‐5p in radiosensitive and radioresistant groups (***P < .001). B, Cutoff value of miR‐18a‐5p in ROC curve. C, ORR in miR‐18a‐5p‐high and miR‐18a‐5p‐low groups (***P < .001). D, Radiation response in miR‐18a‐5p‐high and miR‐18a‐5p‐low groups. E, PFS in miR‐18a‐5p‐high and miR‐18a‐5p‐low groups (*P* = .082). F, OS in miR‐18a‐5p‐high and miR‐18a‐5p‐low groups (*P* = .281)

Totally, 54 eligible patients reached 0 CR, 15 PR, 35 SD, and 4 PD. The median follow‐up was 16.65 months (range: 3.1‐33.6 months) by the deadline of 31 October 2015. Eight patients had no progression, and 19 patients were still alive. We divided all the patients into radiosensitive group (CR+PR, 15 cases) and radioresistant group (SD+PD, 39 cases). The relative expression of plasma miR‐18a‐5p in radiosensitive group was significantly higher than that in radioresistant group (16.02 ± 8.28 vs 1.40 ± 0.72, *P* < .001, Figure 5A). ROC curves demonstrated the cutoff value as miR‐18a‐5p > 2.28 (Figure 5B).

All the patients were divided into miR‐18a‐5p‐high and miR‐18a‐5p‐low groups by the cutoff value of miR‐18a‐5p. The ORR in miR‐18a‐5p‐high and miR‐18a‐5p‐low groups was 86.7% vs 5.1% (*P* < .001, Figure 5C and D). The predictive sensitivity and specificity of plasma miR‐18a‐5p in radiotherapy response reached 87% and 95%, respectively. The median local PFS of miR‐18a‐5p‐high and miR‐18a‐5p‐low groups reached 8.15 months (95% CI: 6.1‐10.1 months) and 6.45 months (95% CI: 5.7‐7.1 months), respectively. There was a trend toward improvement of the median local PFS in miR‐18a‐5p‐high group compared with miR‐18a‐5p‐low group (HR 0.59, 95% CI: 0.34‐1.09, *P* = .082, Figure 5E). The median OS of miR‐18a‐5p‐high and miR‐18a‐5p‐low groups reached 18.90 months (95% CI: 12.2‐25.6 months) and 14.75 months (95% CI: 7.1‐20.3 months), respectively. The median OS from radiotherapy in miR‐18a‐5p‐high group was numerically longer than that in miR‐18a‐5p‐low group (HR 0.82, 95% CI: 0.39‐1.71, *P* = .281, Figure 5F).

Side effects, mainly the RP, were also recorded during the follow‐up. There were 57.4% of grade 1‐4 in all the patients (31/54), 60.0% in radiosensitive group (9/15) and 56.4% in radioresistant group (22/35). There was no significant difference between radiosensitive and radioresistant groups (*P* > .05). There was no significant difference between miR‐18a‐5p‐high and miR‐18a‐5p‐low groups as well (*P* > .05).

## 4 DISCUSSION

In the present study, we successfully induced A549 cells into spheres following the protocols in our previous study.^23^ After sorting, CD133^+^ stem‐like cells expressed higher stemness factors, such as Oct4, Nanog, and Nestin, than did CD133^−^ cells. The subsequent experiments indicated that CD133^+^ cells possessed stemness feature due to their strong sphere forming ability. This model constituted the basis for radioresistance of lung cancer stem‐like cells. Recently, cumulative evidences show the involvement of miRNAs in lung cancer stem‐like cells. However, there is little report on regulatory miRNAs involved in radioresistance of CD133^+^ stem‐like lung cancer cells.

Therefore, we chose miR‐18a‐5p as a regulator of radioresistance of lung cancer stem cells enriched from A549 cells. We found that miR‐18a‐5p was downregulated in A549 cells after radiation and in CD133^+^ stem‐like cells. Then, we examined the biological function of miR‐18a‐5p in radiosensitization of A549 and CD133^+^ stem‐like cells. According to Table 3, CD133^+^ cells were more sensitive to irradiation than parental A549 cells. The radiosensitivity difference between the two cells is caused by the loss of stemness of CD133^+^ cells cultured in IMDM containing 10% calf serum. However, when miR‐18a‐5p was overexpressed in A549 cell and CD133^+^ stem‐like cells, miR‐18a‐5p decreased the D0, Dq, and SF2 values according to the radiation survival curves, suggesting that miR‐18a‐5p overexpression radiosensitized both A549 cells and CD133^+^ stem‐like cells. In vivo experiments showed that miR‐18a‐5p also radiosensitized A549 tumor tissues. Furthermore, the expression of miR‐18a‐5p in xenografts was associated with the survival time of mice. Thus, our study successfully verified that miR‐18a‐5p radiosensitized both A549 cells and CD133^+^ stem‐like cells in vitro and in vivo. To the best of our knowledge, this is the first time that miR‐18a‐5p has been tested in radioresistance of lung cancer stem‐like cells.

Theoretically, miR‐18a‐5p could affect the stemness of lung cancer stem‐like cells. However, miR‐18a‐5p overexpression did not affect the expressions of stemness factors (Oct4, Nanog, and Nestin) and the sphere forming ability of cells, indicating that miR‐18a‐5p was not associated with the stemness of CD133^+^ stem‐like cells.

We further explored the molecular mechanism underlying the radiosensitizing effect of miR‐18a‐5p in lung cancer. As is known, DNA repair after radiation is an important mechanism of radioresistance.^33^, ^34^ Considering that bioinformatic prediction has become a preferential method to explore the targets of miRNAs,^35^, ^36^ we chose potential targets from predicted ones involved in radioresistance and confirmed these targets by biochemical experiments.

Ataxia telangiectasia mutated was proved to be a real target of miR‐18a‐5p in the present study. ATM is a key determinant factor of multiple cellular responses to irradiation, such as DNA double strand break (DSB) repair in cancer cells.^37^, ^38^ Also, ATM could start another important DNA repair of homologous recombination repair (HRR).^39^ DNA damage activates ATM through intermolecular autophosphorylation (phospho S1981) and dimer dissociation.^40^ Inhibition of ATM or pATM (phospho S1981) could radiosensitize cancer cells.^20^, ^41^, ^42^, ^43^ The present study showed that upregulated miR‐18a‐5p inhibited the expressions of ATM and pATM (phospho S1981) and then enhanced the radiosensitivity of A549 cells and CD133^+^ stem‐like cells.

Additionally, we demonstrated that HIF‐1α was regulated by miR‐18a‐5p. Hypoxia is known as a hallmark of solid tumors, which is also a critical cause of radioresistance.^44^ HIF‐1α is an important hypoxia‐responsive transcription factor in tumor microenviroment. Irradiation induces the expression of HIF‐1α, while the latter in turn causes radioresistance by triggering several signaling pathways. HIF‐1α inhibited the apoptosis of malignant cells,^45^, ^46^ induced γH2AX expression and hypoxic condition, enhanced DNA repair and finally led to the poor response of tumor treatment.^47^

As described above, the present study verified two real targets of ATM and HIF‐1α by dual‐luciferase reporter assay and Western blotting. Furthermore, we did a series of experiments to confirm our results. First, we detected the change of γH2AX, a classic DNA damage factor.^48^, ^49^, ^50^, ^51^ Upregulated miR‐18a‐5p suppressed the expression of γH2AX protein, indicating that miR‐18a‐5p inhibited DNA repair and enhanced radiosensitivity. Second, the overexpression of miR‐18a‐5p was found in the miR‐18a‐5p‐LV group before irradiation in the xenografts. The expressions of pATM and HIF‐1α proteins in the xenografts in miR‐18a‐5p‐LV group became lower subsequently.

Consistent with our findings, several previous studies revealed that miR‐18a‐5p was involved in radiosensitivity. For example, miR‐18a‐5p enhanced radiosensitivity via downregulating ATM in cervical cancer,^52^ colorectal cancer,^53^ and breast cancer.^54^ It was addressed that miR‐18 targeted heat shock transcription factor 2 (HSF2),^55^ while HSF2 upregulated HIF‐1α.^56^ Additionally, other studies showed that several miRNAs were associated with DNA repair. For example, miR‐421 and miR‐24 could target ATM^57^ and γ‐H2AX,^58^ respectively. There were also several studies showing that miRNAs were associated with radiosensitivity in lung cancer. Radiotherapy response of lung cancer cells was modulated by miR‐214 through regulation of p38 MAPK, apoptosis, and senescence.^19^ The radiosensitization of lung cancer cells was induced by miR‐122 via targeting BCL‐W and IGF1R.^59^ Enhanced miR‐15a/16 promoted the radiosensitivity of lung cancer via downregulating the TLR1/NF‐*κ*B pathway.^60^ Knockdown of miR‐1323 restored radiosensitivity by suppression of PRKDC activity in radiation‐resistant lung cancer cells.^61^ Our findings added another evidence that miR‐18a‐5p was involved in radiosensitivity in lung cancer cells. More importantly, we demonstrated that miR‐18a‐5p could radiosensitize CD133^+^ stem‐like lung cancer cells.

Several reports showed that circulating miRNAs could be potential biomarkers for early diagnosis and prognosis prediction in lung cancer.^62^, ^63^ Shen et al^64^ found that the expression of miR‐18a‐5p in tumor tissues was correlated with the radiotherapy effect in NSCLC patients. However, there has been no report about the role of plasma miR‐18a‐5p in predicting radiosensitivity in NSCLC patients before.

In the present study, we collected 54 plasma samples from NSCLC patients receiving radiotherapy alone. It is known that concurrent chemoradiotherapy is the standard treatment for patients with stage IIIa‐IIIb (not IV), unresectable lung cancer. Because the patients in the early stage of lung cancer commonly received surgery, patients enrolled in this study were all unresectable (stage III–IV). They rejected concurrent chemoradiotherapy or could not stand this treatment because of clinical reasons, such as huge primary tumor, respiratory and heart malfunction, etc. Every enrolled patient had signed an informed consent before receiving treatment. After 2‐4 cycles of chemotherapy, radiotherapy alone was reasonable. As reported, once the cancer stem cells are cultured in serum medium, they will differentiate into tumor cells and return to the same proportion as was in the source from which they were isolated^65^. In order to exclude the influence of chemotherapy on the enrichment of cancer stem‐like cells and the radiosensitivity, there was an interval of at least two weeks between chemotherapy and radiotherapy. Similar study design has been published recently.^66^ The plasma level of miR‐18a‐5p displayed that miR‐18a‐5p was associated with radiotherapy response in NSCLC patients. We also found that miR‐18a‐5p could be a good radiation indicator with high sensitivity (87%) and high specificity (95%). These results imply that plasma miR‐18a‐5p could be used to stratify NSCLC patients into radiosensitive and radioresistant subgroups. For the radioresistant subgroup, comprehensive intervention should be considered before radiotherapy, such as a higher dose or a concurrent strategy with other anticancer therapies. For the radiosensitive subgroup, a lower dose than standard regimen (60 Gy) could be a possible option. Since tumor size is relevant to the response to radiotherapy, we compared the tumor size between radiosensitive group (82.02 ± 104.09 cm^3^) and radioresistant group (81.01 ± 65.27 cm^3^). The data showed that there was no significant difference (*P* > 0.05). Thus, the difference in radiosensitivity was mainly due to the different miR‐18a‐5p level between the two groups.

Collectively, our study showed that miR‐18a‐5p could function as an indicator of radiosensitivity of lung cancer in vitro, in vivo, and in patients. Our findings here support that miR‐18a‐5p is critical for regulating the DNA repair gene of ATM and hypoxia associated gene of HIF‐1α. As miR‐18a‐5p inhibited both ATM and HIF‐1α in the present study, miR‐18‐5p could be a promising target to improve radiosensitivity in lung cancer. The plasma miR‐18a‐5p could be a novel indicator of radiosensitivity in NSCLC patients. To further confirm the indicator in clinical application, future work of early clinical trial is still needed.

## CONFLICT OF INTEREST

The authors declare that they have no competing interests.

## References

[1] Allemani C , Weir HK , Carreira H , et al. Global surveillance of cancer survival 1995‐2009: analysis of individual data for 25,676,887 patients from 279 population‐based registries in 67 countries (CONCORD‐2). Lancet. 2015;385:977‐1010. 25467588 10.1016/S0140-6736(14)62038-9 PMC4588097

[2] Zarogoulidis K , Zarogoulidis P , Darwiche K , et al. Treatment of non‐small cell lung cancer (NSCLC). J Thorac Dis. 2013;5(Suppl 4):S389‐S396. 24102012 10.3978/j.issn.2072-1439.2013.07.10 PMC3791496

[3] Dessard‐Diana B , Manoux D , Diana C , Housset M , Baillet F . Discussion on the role of radiotherapy in non‐small cell lung cancer apropos of 137 non‐metastatic cases. Cancer Radiother. 1997;1:154‐158. 9273187 10.1016/s1278-3218(97)83533-0

[4] Kaveh K , Kohandel M , Sivaloganathan S . Replicator dynamics of cancer stem cell: Selection in the presence of differentiation and plasticity. Math Biosci. 2016;272:64‐75. 26683105 10.1016/j.mbs.2015.11.012

[5] Krause M , Dubrovska A , Linge A , Baumann M . Cancer stem cells: Radioresistance, prediction of radiotherapy outcome and specific targets for combined treatments. Adv Drug Deliv Rev. 2017;109:63‐73. 26877102 10.1016/j.addr.2016.02.002

[6] MacDonagh L , Gray SG , Breen E , et al. Lung cancer stem cells: The root of resistance. Cancer Lett. 2016;372:147‐156. 26797015 10.1016/j.canlet.2016.01.012

[7] Diehn M , Cho RW , Lobo NA , et al. Association of reactive oxygen species levels and radioresistance in cancer stem cells. Nature. 2009;458:780‐783. 19194462 10.1038/nature07733 PMC2778612

[8] Moncharmont C , Levy A , Gilormini M , et al. Targeting a cornerstone of radiation resistance: cancer stem cell. Cancer Lett. 2012;322:139‐147. 22459349 10.1016/j.canlet.2012.03.024

[9] Sheinerman KS , Tsivinsky VG , Umansky SR . Analysis of organ‐enriched microRNAs in plasma as an approach to development of Universal Screening Test: feasibility study. J Transl Med. 2013;11:304. 24330742 10.1186/1479-5876-11-304 PMC3867418

[10] Srivastava A , Goldberger H , Dimtchev A , et al. MicroRNA profiling in prostate cancer‐the diagnostic potential of urinary miR‐205 and miR‐214. PLoS ONE. 2013;8:e76994. 24167554 10.1371/journal.pone.0076994 PMC3805541

[11] Mari‐Alexandre J , Sanchez‐Izquierdo D , Gilabert‐Estelles J , Barcelo‐Molina M , Braza‐Boils A , Sandoval J . miRNAs regulation and its role as biomarkers in endometriosis. Int J Mol Sci. 2016;17:93. 10.3390/ijms17010093 PMC4730335 26771608

[12] The microRNA Database. https://www.mirbase.org. Accessed July 3, 2014.

[13] Brighenti M . MicroRNA and MET in lung cancer. Ann Transl Med. 2015;3:68. 25992367 10.3978/j.issn.2305-5839.2015.01.26 PMC4402600

[14] Frixa T , Donzelli S , Blandino G . Oncogenic MicroRNAs: key players in malignant transformation. Cancers (Basel). 2015;7:2466‐2485. 26694467 10.3390/cancers7040904 PMC4695904

[15] Rupaimoole R , Calin GA , Lopez‐Berestein G , Sood AK . miRNA deregulation in cancer cells and the tumor microenvironment. Cancer Discov. 2016;6:235‐246. 26865249 10.1158/2159-8290.CD-15-0893 PMC4783232

[16] Hatano K , Kumar B , Zhang Y , et al. A functional screen identifies miRNAs that inhibit DNA repair and sensitize prostate cancer cells to ionizing radiation. Nucleic Acids Res. 2015;43:4075‐4086. 25845598 10.1093/nar/gkv273 PMC4417178

[17] Vincenti S , Brillante N , Lanza V , et al. HUVEC respond to radiation by inducing the expression of pro‐angiogenic microRNAs. Radiat Res. 2011;175:535‐546. 21361781 10.1667/RR2200.1

[18] Asuthkar S , Velpula KK , Nalla AK , Gogineni VR , Gondi CS , Rao JS . Irradiation‐induced angiogenesis is associated with an MMP‐9‐miR‐494‐syndecan‐1 regulatory loop in medulloblastoma cells. Oncogene. 2014;33:1922‐1933. 23728345 10.1038/onc.2013.151

[19] Salim H , Akbar NS , Zong D , et al. miRNA‐214 modulates radiotherapy response of non‐small cell lung cancer cells through regulation of p38MAPK, apoptosis and senescence. Br J Cancer. 2012;107:1361‐1373. 22929890 10.1038/bjc.2012.382 PMC3494421

[20] Yan D , Ng WL , Zhang X , et al. Targeting DNA‐PKcs and ATM with miR‐101 sensitizes tumors to radiation. PLoS ONE. 2010;5:e11397. 20617180 10.1371/journal.pone.0011397 PMC2895662

[21] Garofalo M , Croce CM . Role of microRNAs in maintaining cancer stem cells. Adv Drug Deliv Rev. 2015;81:53‐61. 25446141 10.1016/j.addr.2014.11.014 PMC4445133

[22] Chhabra R , Saini N . MicroRNAs in cancer stem cells: current status and future directions. Tumour Biol. 2014;35:8395‐8405. 24964962 10.1007/s13277-014-2264-7

[23] Lin S , Sun JG , Wu JB , et al. Aberrant microRNAs expression in CD133(+)/CD326(+) human lung adenocarcinoma initiating cells from A549. Mol Cells. 2012;33:277‐283. 22349807 10.1007/s10059-012-2252-y PMC3887701

[24] PicTar. http://pictar.mdc-berlin.de/cgi-bin/PicTar_vertebrate.cgi. Accessed March 26, 2007.

[25] TargetScan Release 5.2. http://www.targetscan.org/vert_50/. Accessed 17 June 2007.

[26] miRanda. http://www.microrna.org/. Accessed 27 August 2010.

[27] Feng ZM , Qiu J , Chen XW , et al. Essential role of miR‐200c in regulating self‐renewal of breast cancer stem cells and their counterparts of mammary epithelium. BMC Cancer. 2015;15:645. 26400441 10.1186/s12885-015-1655-5 PMC4581477

[28] Tiscornia G , Singer O , Verma IM . Production and purification of lentiviral vectors. Nat Protoc. 2006;1:241‐245. 17406239 10.1038/nprot.2006.37

[29] Chen X , Xu Y , Liao X , et al. Plasma miRNAs in predicting radiosensitivity in non‐small cell lung cancer. Tumour Biol. 2016;37:11927‐11936. 27075472 10.1007/s13277-016-5052-8 PMC5080326

[30] Fritz AG , ed. International classification of diseases for oncology: ICD‐O, 3rd edn Geneva: World Health Organization; 2000.

[31] RNAhybrid. http://bibiserv.techfak.uni-bielefeld.de/rnahybrid. Accessed July 6, 2004.

[32] RNAalifold. http://rna.tbi.univie.ac.at/cgi-bin/RNAWebSuite/RNAalifold.cgi. Accessed November 11, 2008.

[33] Ciccia A , Elledge SJ . The DNA damage response: making it safe to play with knives. Mol Cell. 2010;40:179‐204. 20965415 10.1016/j.molcel.2010.09.019 PMC2988877

[34] Borchiellini D , Etienne‐Grimaldi MC , Thariat J , Milano G . The impact of pharmacogenetics on radiation therapy outcome in cancer patients. A focus on DNA damage response genes. Cancer Treat Rev. 2012;38:737‐759. 22387145 10.1016/j.ctrv.2012.02.004

[35] Bentwich I . Prediction and validation of microRNAs and their targets. FEBS Lett. 2005;579:5904‐5910. 16214134 10.1016/j.febslet.2005.09.040

[36] Doran J , Strauss WM . Bio‐informatic trends for the determination of miRNA‐target interactions in mammals. DNA Cell Biol. 2007;26:353‐360. 17504030 10.1089/dna.2006.0546

[37] Xue L , Yu D , Furusawa Y , et al. Regulation of ATM in DNA double strand break repair accounts for the radiosensitivity in human cells exposed to high linear energy transfer ionizing radiation. Mutat Res. 2009;670:15‐23. 19583974 10.1016/j.mrfmmm.2009.06.016

[38] Kastan MB , Lim DS , Kim ST , Yang D . ATM‐a key determinant of multiple cellular responses to irradiation. Acta Oncol. 2001;40:686‐688. 11765061 10.1080/02841860152619089

[39] Morrison C , Sonoda E , Takao N , Shinohara A , Yamamoto K , Takeda S . The controlling role of ATM in homologous recombinational repair of DNA damage. EMBO J. 2000;19:463‐471. 10654944 10.1093/emboj/19.3.463 PMC305583

[40] Bakkenist CJ , Kastan MB . DNA damage activates ATM through intermolecular autophosphorylation and dimer dissociation. Nature. 2003;421:499‐506. 12556884 10.1038/nature01368

[41] Koll TT , Feis SS , Wright MH , et al. HSP90 inhibitor, DMAG, synergizes with radiation of lung cancer cells by interfering with base excision and ATM‐mediated DNA repair. Mol Cancer Ther. 2008;7:1985‐1992. 18645008 10.1158/1535-7163.MCT-07-2104 PMC2671002

[42] Russell KJ , Wiens LW , Demers GW , Galloway DA , Plon SE , Groudine M . Abrogation of the G2 checkpoint results in differential radiosensitization of G1 checkpoint‐deficient and G1 checkpoint‐competent cells. Cancer Res. 1995;55:1639‐1642. 7712467

[43] Zou J , Qiao X , Ye H , et al. Antisense inhibition of ATM gene enhances the radiosensitivity of head and neck squamous cell carcinoma in mice. J Exp Clin Cancer Res. 2008;27:56. 18950535 10.1186/1756-9966-27-56 PMC2584003

[44] Kessler J , Hahnel A , Wichmann H , et al. HIF‐1alpha inhibition by siRNA or chetomin in human malignant glioma cells: effects on hypoxic radioresistance and monitoring via CA9 expression. BMC Cancer. 2010;10:605. 21050481 10.1186/1471-2407-10-605 PMC2992520

[45] Liang D , Yang M , Guo B , Yang L , Cao J , Zhang X . HIF‐1alpha induced by beta‐elemene protects human osteosarcoma cells from undergoing apoptosis. J Cancer Res Clin Oncol. 2012;138:1865‐1877. 22736026 10.1007/s00432-012-1256-5 PMC11824163

[46] Peng XH , Karna P , Cao Z , Jiang BH , Zhou M , Yang L . Cross‐talk between epidermal growth factor receptor and hypoxia‐inducible factor‐1alpha signal pathways increases resistance to apoptosis by up‐regulating survivin gene expression. J Biol Chem. 2006;281:25903‐25914. 16847054 10.1074/jbc.M603414200 PMC3132567

[47] Wrann S , Kaufmann MR , Wirthner R , Stiehl DP , Wenger RH . HIF mediated and DNA damage independent histone H2AX phosphorylation in chronic hypoxia. Biol Chem. 2013;394:519‐528. 23241668 10.1515/hsz-2012-0311

[48] Ivashkevich A , Redon CE , Nakamura AJ , Martin RF , Martin OA . Use of the gamma‐H2AX assay to monitor DNA damage and repair in translational cancer research. Cancer Lett. 2012;327:123‐133. 22198208 10.1016/j.canlet.2011.12.025 PMC3329565

[49] Redon CE , Weyemi U , Parekh PR , Huang D , Burrell AS , Bonner WM . gamma‐H2AX and other histone post‐translational modifications in the clinic. Biochim Biophys Acta. 2012;1819:743‐756. 22430255 10.1016/j.bbagrm.2012.02.021 PMC3371125

[50] Morrison AJ , Highland J , Krogan NJ , et al. INO80 and gamma‐H2AX interaction links ATP‐dependent chromatin remodeling to DNA damage repair. Cell. 2004;119:767‐775. 15607974 10.1016/j.cell.2004.11.037

[51] Lowndes NF , Toh GW . DNA repair: the importance of phosphorylating histone H2AX. Curr Biol. 2005;15:R99‐R102. 15694301 10.1016/j.cub.2005.01.029

[52] Liu S , Pan X , Yang Q , et al. MicroRNA‐18a enhances the radiosensitivity of cervical cancer cells by promoting radiation‐induced apoptosis. Oncol Rep. 2015;33:2853‐2862. 25963391 10.3892/or.2015.3929

[53] Wu CW , Dong YJ , Liang QY , et al. MicroRNA‐18a attenuates DNA damage repair through suppressing the expression of ataxia telangiectasia mutated in colorectal cancer. PLoS ONE. 2013;8:e57036. 23437304 10.1371/journal.pone.0057036 PMC3578802

[54] Song L , Lin C , Wu Z , et al. miR‐18a impairs DNA damage response through downregulation of ataxia telangiectasia mutated (ATM) kinase. PLoS ONE. 2011;6:e25454. 21980462 10.1371/journal.pone.0025454 PMC3181320

[55] Bjork JK , Sandqvist A , Elsing AN , Kotaja N , Sistonen L . miR‐18, a member of Oncomir‐1, targets heat shock transcription factor 2 in spermatogenesis. Development. 2010;137:3177‐3184. 20724452 10.1242/dev.050955

[56] Chen R , Liliental JE , Kowalski PE , Lu Q , Cohen SN . Regulation of transcription of hypoxia‐inducible factor‐1alpha (HIF‐1alpha) by heat shock factors HSF2 and HSF4. Oncogene. 2011;30:2570‐2580. 21258402 10.1038/onc.2010.623

[57] Shin S , Cha HJ , Lee EM , et al. Alteration of miRNA profiles by ionizing radiation in A549 human non‐small cell lung cancer cells. Int J Oncol. 2009;35:81‐86. 19513554

[58] Hu H , Gatti RA . MicroRNAs: new players in the DNA damage response. J Mol Cell Biol. 2011;3:151‐158. 21183529 10.1093/jmcb/mjq042 PMC3104011

[59] Ma D , Jia H , Qin M , et al. MiR‐122 induces radiosensitization in non‐small cell lung cancer cell line. Int J Mol Sci. 2015;16:22137‐22150. 26389880 10.3390/ijms160922137 PMC4613300

[60] Lan F , Yue X , Ren G , et al. miR‐15a/16 enhances radiation sensitivity of non‐small cell lung cancer cells by targeting the TLR1/NF‐kappaB signaling pathway. Int J Radiat Oncol Biol Phys. 2015;91:73‐81. 25442346 10.1016/j.ijrobp.2014.09.021

[61] Li Y , Han W , Ni TT , et al. Knockdown of microRNA‐1323 restores sensitivity to radiation by suppression of PRKDC activity in radiation‐resistant lung cancer cells. Oncol Rep. 2015;33:2821‐2828. 25823795 10.3892/or.2015.3884

[62] Hu Z , Chen X , Zhao Y , et al. Serum microRNA signatures identified in a genome‐wide serum microRNA expression profiling predict survival of non‐small‐cell lung cancer. J Clin Oncol. 2010;28:1721‐1726. 20194856 10.1200/JCO.2009.24.9342

[63] Shen J , Todd NW , Zhang H , et al. Plasma microRNAs as potential biomarkers for non‐small‐cell lung cancer. Lab Invest. 2011;91:579‐587. 21116241 10.1038/labinvest.2010.194 PMC3130190

[64] Shen Z , Wu X , Wang Z , Li B , Zhu X . Effect of miR‐18a overexpression on the radiosensitivity of non‐small cell lung cancer. Int J Clin Exp Pathol. 2015;8:643‐648. 25755757 PMC4348909

[65] Gupta PB , Fillmore CM , Jiang G , Shapira SD , Tao K, Kuperwasser C , Lander ES . Stochastic state transitions give rise to phenotypic equilibrium in populations of cancer cells. Cell. 2011;146:633‐644. 21854987 10.1016/j.cell.2011.07.026

[66] Maquilan G , Grover S , Xanthopoulos E , Evans TL , Aggarwal C , Langer CJ , Cohen RB , Stevenson JP , Simone CB , Rengan R . Analysis of the Relationship Between Response to Chemotherapy and Response to Radiation Therapy in Patients With Non‐Small Cell Lung Cancer Receiving Sequential Treatment. Am J Clin Oncol. 2018;41:391‐395. 27100960 10.1097/COC.0000000000000288

